# Prevalence and Pattern of Road Traffic Accidents among Commercial Motorcyclists in the Central Tongu District, Ghana

**DOI:** 10.1155/2020/9493718

**Published:** 2020-06-01

**Authors:** Kennedy Diema Konlan, Abdul Razak Doat, Iddrisu Mohammed, Roberta Mensima Amoah, Joel Afram Saah, Kennedy Dodam Konlan, Juliana Asibi Abdulai

**Affiliations:** ^1^Department of Public Health Nursing, School of Nursing and Midwifery, University of Health and Allied Sciences, Ho, Volta Region, Ghana; ^2^Tehran University of Medical Sciences, School of Nursing and Midwifery, Tehran, Iran; ^3^Nurses' and Midwives' Training College, Tamale, Northern Region, Ghana; ^4^Department of Public Health, School of Allied Sciences, University for Development Studies, Tamale, Northern Region, Ghana; ^5^Department of Nursing, West End University College, Accra, Ghana; ^6^Department of Surgery, Tamale Teaching Hospital, Tamale, Northern Region, Ghana

## Abstract

**Background:**

The World Health Organisation estimates that 1.35 million people die as a result of road traffic crashes. Motorcycles as a means of transport are increasingly becoming the preferred and easiest means of transportation for most people in developing countries despite the associated risk. This study determined the prevalence and pattern of motorcycle crashes in Adidome among commercial motorcyclists.

**Methods:**

A descriptive, cross-sectional study design was used as 114 commercial motorcyclists were recruited to respond to a pretested research questionnaire in the Adidome district of the Volta Region. Data were analyzed using SPSS, version 22.0. Data were presented as simple descriptive statistics. A chi-square relationship was determined using the demographic variables, and the history of accident at a 95% confidence interval with 0.05 was considered as statistically significant.

**Results:**

The prevalence of road traffic crashes at Adidome was 64.0%. Motorcyclists (74.0%) were reported to have been involved in crashes in the past one year prior to the study. Motorcyclists attributed the last accident to excessive speeding (31.5%) and bad roads (23.3%), this accident as a result of colliding with another motorcycle (50.7%), and slippery surfaces (24.7%). The majority (63.0%) of the respondents had an accident once. The consumption of alcohol was associated with the occurrence of an accident as 34.2% occurred among cyclists who drank alcohol, compared with 29.8% who did not (*p* < 0.05).

**Conclusion:**

There should be strict implementation of current road traffic regulations of Ghana by the MTTD of the Ghana Police Service, and penalties should be awarded against anybody caught riding a motorcycle under the influence of alcohol. Helmet and other protective devices must be made compulsory for motorcycle riders to prevent injuries, especially head injuries, if an accident occurs.

## 1. Introduction

The World Health Organisation (WHO) estimates that 1.35 million people die as a result of road traffic crashes [1]. Between 20 and 50 million more people suffer nonfatal injuries, with many, incurring a disability as a result of road traffic accidents [1]. Road traffic injuries cause significant economic losses to individuals and their families and countries all over the world. These losses may be associated with cost of treatment, loss of productivity and valuable working time for victims and relatives, loss of skilled labour force, and loss of school hours. Road traffic crashes cost most countries 3% of their gross domestic product [1]. Road traffic injuries are now the leading killer of people aged 5–29 years. The burden is disproportionately borne by pedestrians, cyclists, and motorcyclists, in particular those living in developing countries. It is further estimated that 770 million motorcycles are on the roads worldwide and engage in various activities. In the developing world, motorcycles are used in serviceable responsibilities related to mobility, transport, sport, and economic activities [2]. Motorcyclists' accidents denote more than 380,000 annual deaths worldwide and 28% of the global fatalities on the roads in 2016. More than 90% of road traffic deaths occur in low- and middle-income countries, making Africa generally more vulnerable. Road traffic injury death rates are highest in the African region [1]. Even within high-income countries, people from lower socioeconomic backgrounds are more likely to be involved in road traffic crashes.

Road transport is the dominant mode of transport in Ghana [1]. It accounts for more than 80% of passenger traffic and over 70% of freight traffic in the country [1]. Increased economic activities and investments in road transport infrastructures have resulted in increased usage of motorcycles, more especially in the urban centres where it is used for commercial purposes though their operations are illegal [3]. Yet increased use of motorcycles has been accompanied by an unprecedented increase in road traffic crashes. In Ghana and some other developing countries, motorcycles as a means of transport is increasingly becoming the preferred and easiest means of transport for most people despite the associated risk [4], an average of 3,242 people lose their lives due to road traffic injury (RTI) every day [1]. It is estimated that 1.35 million lives are lost through road accidents each year with as many as 50 million injuries occurring [1]. Road traffic deaths were projected to become the third most important health problem by 2020, a phenomenon which could be attributed to increased modernization in many developing countries [5]. Commercial motorcycling has become a popular mode of transportation in both rural and urban areas of developing countries since the early 1990s, but with its antecedents traced to 1960s. Its related injuries cause significant morbidity and mortality. Many road users such as motorists and pedestrians among others see their presence on the roads as the cause of congestion, confusion, fear, and decreased safety in the road system [6]. In urban areas, the use of motorcycles for commercial purposes popularly known as “Okada” has increased due to rising unemployment despite the fact that their operations are illegal per the laws of Ghana and coupled with its associated risk to commuters.

Motorcycle injuries constitute a major but neglected emerging public health problem in developing countries and are one of the leading causes of injuries and deaths among victims of accidents [3]. There have been varying accounts of the spate of road traffic accidents all over the world; from 22.8% in China to as high as 62% in Vietnam [6]. In sub-Saharan Africa, the use of commercial motorcycles by young people in the business of transportation has become quite rampant in recent times and its popularity and widespread acceptance has rapidly inched up in recent years due to the fact that it has become a means of gainful employment to quite a number of people following the increase in the rate of unemployment [4]. Several factors account for this high spate of use of commercial motorcycles for transportation. These factors include inadequate mass transport system, bad roads, and traffic congestion among others in developing countries [7]. Again the ability of these motorcyclists to manoeuvre their way through heavy traffic jams has made it the quickest mode of transportation as other means of transport such as cars are unable to access these roads. The risk of being involved in a road accident is over eight times as great for a motorcycle as for a car; a motorcyclist is 24 times more likely to be killed or seriously injured per kilometre journey than a car driver [8]. Road crashes kill an average of four people daily in Ghana. In 2005, the number of road crashes increased by 16% relative to 2004 [9]. Road users aged between 16 and 45 years are the most vulnerable group and account for 58% of total road crash fatalities from 2002 to 2005. 70% of persons killed in road crashes are males. The age groups 0–5, 46–65, and over 65 years also accounted for 20.8%, 16.7%, and 4.6%, respectively, of the total fatalities during the same period in Ghana [9]. An annual distribution of fatalities by road user crash hovers around 3.5% of the motorcyclists involved in fatalities. These fatalities were reportedly mostly due to head injuries, which could have been preventable if motorcyclists were wearing crash helmets [10].

A commercial motorcyclist is described as a person who uses a motorcycle to transport people from place to place for the purpose of receiving money. This category of motorcyclists can also be classified as motorcycle taxis. Commercial motorcyclists ply their trade in the informal sector and have no any identifiable group that regulates specifically their activities. The lack of adequate and sustainable public transport coupled with poor urban planning in most urban sectors in Ghana resulted in a transport gap. Hence in Ghana, motorcyclists have therefore exploited motorcycles to transport people from place to place for money (commercial motor taxi). Motorcycle use as a means of transportation in Nigeria heightened due to its convenience, affordability, easy manoeuvrability, and ability to navigate through poor road networks and traffic congestions found in large and commercial cities, compared with four-wheeled vehicles [11]. Health care authorities as well as metropolitan and district authorities are investing a lot of resources in campaigns towards attaining an accident-free society especially in Ghana [12]. Reports in most metropolises in Ghana indicate that at least a crash helmet motorcycle rider is killed every fortnight and the number of motorcycle injuries has reportedly increased in recent times [13]. Ghana estimated road traffic accidents to cost 1.6% of gross domestic product (GDP), which translated to US$ 165 million [14]. The National Road Safety Commission (NRSC) [14] also noted that motorcycle accidents accounted for 4% of all road traffic accidents in the country.

### 1.1. Problem Statement

**Figure 1:**
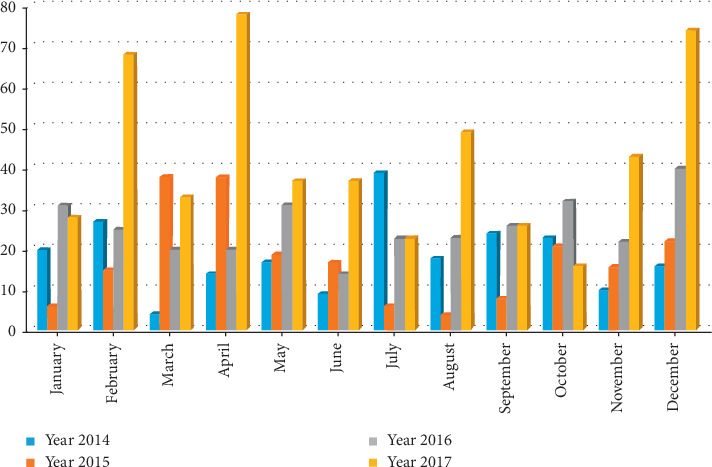
Report of road traffic crashes at Central Tongu from 2014 to 2017 [16].

Several factors have been associated with motor riders having a high incidence of crashes, especially in urban and peri-urban communities where it is used as a means of transport. Motor riders ride without a prior notion about crashes in their mind, hence risking their lives by riding while unprotected or under the influence of excessive alcohol [4]. According to data compiled by the Ghana Motor Traffic and Transport Department [15], a total of 2,076 people died in motorcycle traffic accidents in 2017, with a total number of 3,487 motorcycles being involved, and in the Volta Region having 823 people dying [6]. The occurrence of road traffic crashes associated with commercial motorcycling has also been on the increase with a rise in the number of injuries presented at hospitals as shown in Figure 1. Even though motorcyclists are at high risk of road traffic crashes, few studies have been done on that with only a few community-based studies done on the operations of motorcyclists in Ghana. Adidome, a district capital of Central Tongu in the Volta Region, has seen similar increasing emergence of commercial motorcycles as a major source of transportation for the people in the area. Several motorcycle crashes have been recorded in the area, but there is no current research that investigates the prevalence and pattern of injury among these commercial motorcyclists. Conducting a study of motorcycle crashes in this setting will unravel the possible causes as well as possible solutions, which will enable authorities in charge of the district take concrete measures to help reduce the spate of road traffic crashes in the district.

### 1.2. Aim

This study determined the prevalence and pattern of road traffic crashes among commercial motorcyclists in Adidome, the Central Tongu District of the Volta Region (i.e., motorcycle taxi drivers).

## 2. Methodology

### 2.1. Research Design

A descriptive cross-sectional design was used. The researchers, using a questionnaire, assessed the perception of commercial motorcyclists in the Central Tongu District of the Volta Region where commercial motorcycling is emerging as a major means of transport for residents.

### 2.2. Study Setting

The study was conducted at Adidome, which is the capital of the Central Tongu District in the Volta Region of Ghana. The Volta River flows close to the town south ways towards the Atlantic Ocean. Because of the closeness of the river and nature of the soil, the people in the town and surrounding villages are engaged in farming and fishing, whilst some of the women are engaged in pottery and other activities such as kente weaving. The population of Adidome according to the 2010 Population and Housing Census is 7,587, representing 12.8% of the district's total population, out of which 3,377 (44.5%) represents the population of male, while female constitutes 55.5% with an absolute value of 4,210. The town has one hospital and a private clinic (Biodum Maternity) that helps health service delivery. There are other health facilities in the nearby towns, which supplement health services in the district. The major means of commuting of residents include the use of roads and the river that connect the town to other communities. Also until recently, residents had to trek on foot from place to place. Lately, commercial motorcycling has become a common means of transport and has provided jobs to many youths who hitherto were unemployed. This particular means of transport is increasingly becoming popular as it provides low cost, convenient means of transport.

### 2.3. Study Population and Sampling

The study population for this study was commercial motorcyclists in the Adidome Township. A commercial motorcyclist was described as a person who uses a motorcycle to transport people from place to place for the purpose of receiving money. This category of motorcyclists can also be classified as motorcycle taxis. Commercial motorcyclists ply their trade in the informal sector and have no any identifiable group that regulates specifically their activities. This population is largely a fluid population as there was no identifiable grouping or registration to commence business. Any person in possession of a motorcycle could engage in the business. However, people who engage in this business usually have a designated place where they wait for passengers. The study population, therefore, included all these persons who were located at these vantage points and engaged in commercial cycling. The locations at which passengers could be gotten were seven in the district. At each location, a self-acclaimed leader estimated the number of commercial motorcyclists. In total, 172 commercial motorcyclists were identified in the town.

The sample size was determined using the Yamane formula:

(1) $$\begin{array}{c} n=\frac{N}{1+N\left(e^{2}\right)}, \end{array}$$

where *n* = sample size, *N* = population size, and *e* = margin of error (0.05):

(2) $$\begin{array}{c} n=\frac{172}{1+172\left(0.05^{2}\right)}=120.2797\\ n=120. \end{array}$$

The sample was then proportionally divided, so that each grouping was well represented. Within each cluster, respondents who were readily available and willing to respond to the questionnaire were recruited for the study. All commercial motorcyclists who were contacted to respond to the questionnaire gave consent to participate.

### 2.4. Data Collection and Analysis

The data were collected between February and June 2018. Prior to data collection, five research assistants were recruited and trained in basic ethics of data collection and on the study instrument. The research assistants had a minimum of a bachelor's degree. Research assistants were designated where commercial motor riders usually park to carry passengers to recruit respondents. It took an average of 15 minutes to complete each questionnaire. The instrument for data collection was an interviewer-administered questionnaire consisting of 21 question items divided into five sections. Section A of the questionnaire comprises the demographic characteristics of the respondents such as age, gender, educational level, and marital status. Section B determined the rate of RTA among commercial motorcyclists. Section C determined the patterns and degrees of injuries among commercial motorcyclists. Section D explored the factors associated with RTA among commercial motorcyclists, while section E assessed the awareness level of the respondent on traffic regulations. Pretesting of instruments was done at Battor, a town in the North Tongu District, after which necessary changes to the questionnaire was made before the main study. The changes made after pretesting gave clarity to questions that were ambiguous. A total of 10 motorcyclists were recruited for the pretesting.

Completed questionnaires were collected by the researchers, kept and filed without exposing the information of the respondents. The data collected were analyzed using SPSS, version 22.0. It was presented in descriptive form such as frequency and proportion. Also cross-tabulations were done to establish some relationships. A chi-square of relationship was determined using the demographic variables, and the history of accident at a 95% confidence interval with 0.05 was considered as statistically significant.

### 2.5. Ethical Consideration

Ethical clearance was obtained from the Research Ethics Committee of the University of Health and Allied Sciences, UHAS (UHAS-REC A.10 [10] 17–18). Permission was also obtained from the district health management authorities, the district health management team, as well as from the Road and Safety Commission for the collection of data in the district. Respondents were recruited into the study after they agreed and signed a written consent form that contained the purpose, objectives, and significance of the study.

## 3. Results

A total of one hundred and fourteen male commercial motorcyclists responded to a structured questionnaire. The age distribution showed 10.5% were below the age of 20 years, 57.9% of respondents were between the ages of 20–30, 26.3% of the respondents were between the ages of 31–40, and 5.3% were above 40 years. The majority (63.3%) of respondents were single, and 4.4% of the respondents had divorced. Also, 65.8% of the respondents do not drink alcohol.

In relation to their educational level, 50.9% had basic education, 35.1% had secondary education, 5.3% had tertiary education, and 8.8% had no form of formal education. The results show that 64.0% commercial motorcyclists have a history of crashes, while 36% have never been involved in an accident as shown in Table 1. The majority (63.0%) of the respondents had an accident once, 27.4% of the motorcyclists had RTA twice, 8.2% respondents had crashes thrice, and 1.4% of the respondents had an accident four times and more. The majority (74.0%) of those who suffered met with an accident that occurred in the past year. Among them, more than half (72.2%) had had only one accident, 24.1% of the motorcyclists had accident twice, and 3.7% had crashes thrice. A greater proportion (64.0%) of the respondents had been involved in road traffic crashes since they started riding motorcycles. In an episode of an accident, 83.6% of the motorcyclists sustained injuries, while 16.4% had no injuries. The majority (50.7%) of the respondents indicated that they have been involved in crashes by colliding with a vehicle such as cars, trucks, and cargo, 24.7% had an accident as a result of colliding with animals and slippery surfaces of the road, and 6.8% were involved in crashes by colliding with other motorcyclists as in Table 2. Describing the nature of injuries sustained, the majority (54.1%) indicated having injuries on the lower limbs, 23.0% sustaining injury on their upper limbs and 9.8% on their trunk, and 29.2% sustaining their injuries on the head and face. Most (43.8%) of the respondents indicated carrying one passenger during the accident, while 32.9% carried two passengers. The total number of passengers carried by the respondents on their motorbikes during the crashes was 56. 85.7% sustained injuries; however, 14.3% did not. Among the passengers who sustained injuries, most (56.3%) had injuries on their lower limbs, 29.2% sustained injuries on the face and head, and 14.6% having their injuries on the upper limbs.

The reasons for the high spate of crashes included over speeding (31.5%), bad roads (23.3%), wrongful overtaking (17.8%), nonobservant of traffic laws (13.7%), reckless riding (8.2%), and making a phone call while riding (5.5%). Motorcyclists knew about motorcycling regulations (90.4%) as shown in Table 3. Despite the number of respondents who knew about motorcycling regulations, a little more, 107 (93.6%), said they knew they are supposed to register their motorbike, but ironically only a few, 22 (20.6%), registered their motorbike. Motorcyclists were aware that they were supposed to have a licence before riding a motorbike (94.7%), but only 7.4% of the respondents said that they had a licence. Motorcyclists were aware they are supposed to wear a helmet (96.5%), but only 43.6% wear a helmet when riding a motorbike. In terms of knowledge of the traffic act, 71 (62.3%) stated that they have heard about the Road Traffic Act. The majority (85.1%) said they were aware of the existing traffic signs when riding a motorcycle. 94 (82.5%) of the respondents also said they were aware of the existence of signs for pedestrian crossing (82.5%).

The involvement in an accident against the age distribution (*χ*^2^ = 3.124, *p* > 0.05) and marital status (*χ*^2^ = 3.229, *p* > 0.05) were not statistically significant as in Table 4. The prevalence of crashes was higher among those who have attained basic education (38.6%) as compared with those who have secondary (14.0%), tertiary (4.4%), and no education (7.0%) (*χ*^2^ = 15.633, *p* < 0.05). Prevalence of accident was higher among those who drank alcohol (34.2%) than among those who did not (29.8%) (*χ*^2^ = 33.294, *p* < 0.05).

## 4. Discussion

This study assessed the prevalence and pattern of road traffic crashes among commercial motorcycle operatives in the Adidome Township of the Volta Region. Commercial motorcycling has become a common form of employment in recent times among many youths within the town. The prevalence of road traffic crashes among motorcyclists in the present study is 64.0%. This high spate of crashes among commercial motorcyclists within the catchment area of this study manifests the wanton dissipation of life and property as many are left dead or with morbidity. These findings may not however be isolated as similar findings were reported in another jurisdiction; 62.0% in Vietnam, 68.0% in southern Nigeria [8], and 56.1% in India [17]. However, it was higher compared with 25% in Uganda, 22.8% in China, 45.3% in western Nigeria [8], and 18% in Benin City of Nigeria [18]. Also in Rwanda, 38.7% of surveyed motorcycle drivers experienced a crash during their lifetime [19]. The level of risk exposed by commercial motorcyclists is different in different geographical conditions as well as the level of enforcement of local and national laws that protect riders, pillion riders, and other road users. While in the Adidome area, laws are sporadically enforced and the continued observance of these laws is not general throughout the year. The majority (74.0%) of commercial motorcyclists' accidents occurred within the last year. These findings remain worrying as one could immediately deduce that motorcycle crashes are so prevalent and technically higher than the records reported by hospital authorities as well as the National Road Safety Commission. Johnson [8] reported only 21.8% of crashes within a year were among cyclists.

Among commercial motorcyclists who responded to have had an accident (32.9%) were carrying more than one passenger at the time of the accident. This is in clear violation of the National Road Safety Dictates of Ghana that mandates that motorcycles can carry only one pillion rider. This act of having more than one pillion rider is widespread and was reported by Rosenbloom et al. [20] that commercial motorcyclists carry more than one passenger. Consistent with previous studies, educational background and alcohol use were identified as factors that influenced the rate of occurrence of crashes among motorcyclists in this study. Driving under the influence of alcohol and any psychoactive substance or drug increases the risk of a crash that results in death or serious injuries [1]. In northern Ghana, alcohol use was related to accident occurrence as 8.84% of drivers reported consuming alcohol prior to the collision [21]. Also in a descriptive study of autopsy reports of road traffic accidents, Papalimperi et al. [22] reported that 40.7% of the RTA-related fatalities were associated with alcohol consumption [22]. The rate of crashes was higher among participants who have attained basic education (38.6%) as compared with those who have attained higher levels of education. Education remains a key component as it enables road users to read and comprehend basic road signs and regulations. These regulations are inherently protective. Poor knowledge of traffic code and the desire to generate more money were also found to be a significant factor responsible for high rates of accidents among commercial motorcyclists [23]. Also, the rate of accidents was higher among those who drank alcohol (34.2%) than those who did not (29.8%). This indicates the impact of alcohol use on the overall carnage related to road traffic crashes. In Congo, however, it was reported that driving under the influence of alcohol (9%) was the main cause of RTA occurrence [24].

Collision with vehicles such as cars, trucks, and cargo in the region was of 50.7%. Relatively, commercial towns always have cargo and other vehicles packed on the shoulders of the road and sometimes do not have any directional signs for road users or even if available are sometimes inadequate. Odiwuor et al. [6] reported that 60% of the motorcyclists who had accidents reportedly collided with other motorcyclists. Owoaje [25] reported that the collision of a motorcyclist with other motorcyclists was 27.9%. The findings also showed that 24.7% of road crashes were caused by stray animals crossing the road at undesignated places or where there was no signage. In Kenya, motorcyclists (both riders and passengers) as well as pedestrians were found to be the cause of motorcycle accidents [26]. However, Bachani et al. [26] blamed a majority of motorcycle accidents were due to motorcyclists. Low-income countries require solutions to a major public health problem that involve the use of motorcycles and its related challenges. Several studies indicate that low-income countries are affected by high motorcyclist traffic, bad road conditions, and road culture problems [27, 28].

Findings from this study revealed that commercial motorcyclists (83.6%) sustained injuries during the most recent accident. These injuries were on the lower limbs (54.1%). Injuries to the lower extremities have been noted by many other studies as one of the most common injuries associated with motorcycle crashes. This may be associated with the high velocity impact that is usually associated with such crashes. The findings of this study are corroborated by a study conducted in Singapore, where wounds, fractures, and/or dislocations of the limbs were significantly more common among motorcyclists in emergency departments compared with other motor vehicle incidents [29]. Also in Obafemi Awolowo Teaching Hospital, Ile Ife, Nigeria, 79.3% of motorcycle accident victims had limb injuries. The lower limb injuries were also reported in several other studies [8, 30]. This trend of lower limb injuries may be related to the fact that the limbs are often squeezed between the motorcycle and impacting vehicle, the ground, or some other fixed object. Motorcyclists who had an accident in this study had one (43.8%) or two (32.9%) pillion riders at the time of the accident. This makes the likelihood of impact of mortality and morbidity associated with the event even high. However, Johnson [8] reported that 36.5% of the respondents carried one passenger and 39.0% carried more than one passenger during road traffic crashes.

In this study, it was identified that over speeding (31.5%), bad roads (23.3%) wrongful overtaking (17.8%), were the leading causes of crashes. All these factors associated with crashes by motorcyclists are mainly human related, and if regulatory authorities put in appropriate measures, the spate of road traffic crashes will drastically reduce. Odero [10] stated that over speeding, wrong overtaking, and bad roads among others are the factors that influence the rate of accidents among commercial motorcyclists. The impact of over speeding as a cause of road traffic crashes has been well documented. The effect of speeding has shown that even a 1 km/h increase in vehicle speed can lead to as much as a 3.0% increased risk of a crash resulting in an injury [25]. Over speeding is still characteristic of commercial motorcyclists, coupled with the lack of a comprehensive structure in place to ensure motorcyclists comply with stipulated speed limits. These and many more such as poorly designed roads have kept RTA and its related injuries among commercial motorcyclists constantly high. The results of this study showed that making a phone call while riding (5.5%) accounted for some of the crashes that occurred in the area. This finding was similarly reported by Olubiyi et al. [31] that more than three quarters (87%) of the respondents use their mobile phones while driving as the majority (70%) were aware of the possible hazards or risk associated with using mobile phones while driving [31]. Cellular phones decrease a driver's ability to avoid a collision caused by other drivers [32] or other stationary objects or stray animals. Experimental results also suggested that participants engaged in a process of risk compensation, with driving speed being slower at times of a mobile phone conversation [33]. There was also some evidence that the use of a handheld mobile phone (when compared with a hands-free system) was associated with poorer driving performance [33].

Commercial motorcyclists in the Adidome district knew about road traffic regulations (90.4%), the use of helmets (96.5%), acquisition of a licence (94.7%), and registration of their bikes (93.9%). These views were, however, different from the actual practice of the motorcyclists as they indicated 43.6% wore helmets, 20.6% registered their bikes, and 7.4% had licence to ride a motorcycle. This is a clear manifestation of the lack of enforcement of road traffic regulations that are supposed to protect the rider as well as the road users. Even more important are the regulations for those engage in commercial motorcycling because users may not be aware of the dangers associated with the use of their services. The National Road Safety Commission in collaboration with the driver and vehicle licensing authorities must implement pragmatic measures to help curtail this blatant disregard.

The Road Traffic Act of Ghana [34] (ACT 683) stipulates that no person shall drive a motor vehicle on a road unless he holds a valid professional, private, or learner driver's licence authorizing him to drive that class of motor vehicle. The regulation further stated that no person (whether the driver or a passenger) shall ride on a motorcycle unless he is wearing a crash helmet. A study conducted by Boldol and Zalat (2018) in Egypt shows that out of 319 motorcyclists, the majority, 278 (87.1%), did not have a riding licence; with regard to helmet use, only 6 (1.9%) wear. The study by Ofonime (2012) in Ikot Ekpene, southern Nigeria, involving 200 male commercial motorcyclists, showed the majority, 125 (91.9%), were not wearing a helmet when the accident occurred. Adekunle et al. [35] in Sagamu, southern Nigeria, indicated that 9 (3%) out of 300 respondents mostly use helmets when riding their motorbike. This describes a significant disregard for national laws that enjoins all motorcyclists to acquire a driving licence before plying their trade. In Kenya, it is reported that some motorcyclists were unlicensed and rode under the influence of alcohol or other drugs. “Boda boda” injuries accounted for 25% of all cases, pedestrians and motorcyclists were injured in the 78% of “boda boda” accidents [36]. The lack of rider's licence by a commercial cyclist is widespread in Africa as Nasong'o (2015) [36] reported that 58% did not have a valid driving licence, while only 42% had them.

These differences in the rate of helmet use reflect differences in awareness of the role of helmets in preventing or reducing the severity of head injury during motorcycle crashes between these countries and poor enforcement of traffic laws. It could also be due to difference in attitudes to helmet wearing between these countries [37]. In terms of the respondent's awareness of the traffic act and existence of traffic signs, the data indicate that 71 (62.3%) of the respondents have heard about the traffic act and 94 (82.5%) were aware of the existence of traffic signs when riding a motorcycle. Boldol and Zalat (2018) in Egypt among motorcyclists stated that about 185 (58%) out of 319 motorcyclists do not respect traffic rules and traffic lights or road signs, therefore resulting fatal crashes. These discrepancies in regard to knowledge and actual implementation of road regulations as stipulated in national, regional, and district laws and policies must be immediately controlled to reduce the high incidence of road traffic crashes especially among commercial motorcyclists.

## 5. Conclusion

The study revealed that over speeding, reckless riding, nonobservance of traffic laws, wrongful overtaking, and bad roads accounted for the higher percentage of factors that increase the higher rate of crashes among commercial motorcycle riders in Adidome. The study showed that the commercial motorcycle riders do not comply with road safety highway regulations. This is because motor riders carry two or more passengers at a time, do not use safety equipment like a helmet, and do not ride with their rider's licence. Drunk riding, lack of adherence to the highway safety rules, and lack of understanding of the road traffic regulations had a significant contribution in the pattern of road traffic crashes in the area. From the study, it has also been deduced that the motorcycle riders were aware of the rules and regulations governing the road users, but a lesser number really applied their knowledge. This might lead to the prevalence of crashes among commercial motorcyclists in Adidome. Funds should be made available by the government for the creation of road safety awareness using different mediums of communication. The government should invest in improving the road network in Ghana as most accidents are said to be associated with poor roads. There should be strict implementation of current road traffic regulations of Ghana by the MTTD of the Ghana Police Service, and penalties should be awarded against anybody caught riding a motorcycle under the influence of alcohol. Helmet and other protective devices must be made compulsory for motorcycle riders to prevent injuries, especially head injuries, if an accident occurs.

## Figures and Tables

**Table 1:** Demographic characteristics and prevalence of road traffic crashes in Adidome.

Variables	Parameter	Frequency	Percentage

Age distribution	Less than 20	12	10.5
	20–30	66	57.9
	31–40	30	26.3
	More than 40	6	5.3

Sex distribution	Male	114	100.0
	Female	0	0.0

Marital status	Single	72	63.2
	Married	37	32.5
	Divorced	5	4.4

Alcohol consumption	Yes	39	34.2
	No	75	65.8

History of accident	Yes	73	64.0
	No	41	36.0

Number of times involved in an accident	Once	46	63.0
	Twice	20	27.4
	Thrice	6	8.2
	Four times or more	1	1.4

Suffered an accident within the past year	Yes	54	74.0
	No	19	26.0

Frequency of accident within the last year	Once	39	72.2
	Twice	13	24.1
	Thrice	2	3.7

**Table 2:** Patterns and degrees of injuries among commercial motorcyclists.

Variables	Parameter	Frequency	Percentage

Object of collision	Another motorbike	5	6.8
	A stationary object	13	17.8
	A vehicle (car, truck, etc.)	37	50.7
	Others (stray animals)	18	24.7

Sustained injury	Yes	61	83.6
	No	12	16.4

Site of injury	Lower limb	33	54.1
	Upper limb	14	23.0
	Head/face	8	13.1
	Trunk	6	9.8

Number of pillion riders during accident	None	17	23.3
	One	32	43.8
	Two	24	32.9

Pillion riders sustained injury	Yes	48	85.7
	No	8	14.3

Nature of injury of pillion rider	Lower limb	27	56.3
	Upper limb	7	14.6
	Head/face	14	29.2

**Table 3:** Motorcyclist level of knowledge of traffic regulations.

Variables	Parameter	Frequency	Percentage

Awareness of cycling regulations	Yes	103	90.4
	No	11	9.6

Awareness of required to register motorcycle	Yes	107	93.9
	No	7	6.1

Registered motorcycle	Yes	22	20.6
	No	85	79.4

Awareness of required to have riding licence	Yes	108	94.7
	No	6	5.3

Have riding licence	Yes	8	7.4
	No	100	92.6

Awareness to wear crash helmet	Yes	110	96.5
	No	4	3.5

Wears a crash helmet	Yes	48	43.6
	No	62	56.4

Awareness of the Road Traffic Act	Yes	71	62.3
	No	43	37.7

Awareness of existing traffic signs	Yes	97	85.1
	No	17	14.9

Awareness of existing sign of pedestrian crossing	Yes	94	82.5
	No	20	17.5

**Table 4:** Association between respondents' characteristics and involvement in accident.

Variables	Responses	Ever been involved in an accident				
	Parameter	Yes	No	Total	Statistics	*p*value
		73 (64.0)	41 (36.0)	114 (100.0)		
Age	Less than 20	9 (7.9)	3 (2.6)	12 (10.5)	3.124	0.373
	20–30	43 (37.7)	23 (20.2)	66 (57.9)		
	31–40	19 (16.7)	11 (9.6)	30 (26.3)		
	More than 40	2 (1.8)	4 (3.5)	6 (5.3)		

Marital status	Single	42 (36.8)	30 (26.3)	72 (63.2)	3.229	0.199
	Married	28 (24.6)	9 (7.9)	37 (32.5)		
	Divorced	3 (2.6)	2 (1.8)	5 (4.4)		

Educational level	None	8 (7.0)	2 (1.8)	10 (8.8)	15.633	0.001^*∗*^
	Basic	44 (38.6)	14 (12.3)	58 (50.9)		
	Secondary	16 (14.0)	24 (21.1)	40 (35.1)		
	Tertiary	5 (4.4)	1 (0.9)	6 (5.3)		

History of alcohol use	Yes	39 (34.2)	0 (0.0)	39 (34.2)	33.294	0.001^*∗*^
	No	34 (29.8)	41 (36.0)	75 (65.8)		

## Data Availability

All data sets from which the conclusion of this manuscript is based have all been stated in this manuscript, and there are no data deposited in any data repositories elsewhere.
